# Design of organic structure directing agents to guide the synthesis of zeolites for the separation of ethylene–ethane mixtures†

**DOI:** 10.1039/d0ra02896g

**Published:** 2020-05-27

**Authors:** Frits Daeyaert, Michael W. Deem

**Affiliations:** Department of Bioengineering, Rice University 6100 Main St Houston TX USA mwdeem@rice.edu; FD Computing Stijn Streuvelsstraat 64, 2340 Beerse Belgium; Department of Physics & Astronomy, Rice University 6100 Main St Houston TX USA

## Abstract

Industrial production of ethylene entails a costly separation from the ethane by-product, and this separation is the dominant consumer of energy in the process. Zeolites have been proposed as a next generation material for this separation process, and a molecular screen of all known zeolites has revealed several promising candidate materials. None of the identified materials has yet been synthesized in the all-silica form evaluated in the screen. We here design organic structure directing agents (OSDAs) for four of the zeolites with the best predicted separation performance, two that are ethylene selective and two that are ethane selective. The designed OSDAs may enable the synthesis of these zeolites for more energy efficient separation of ethylene and ethane.

## Abstract 2

Structure directing agents were designed in this work to aid in the synthesis of four zeolites predicted to efficiently separate ethane and ethylene.

## Graphical abstract

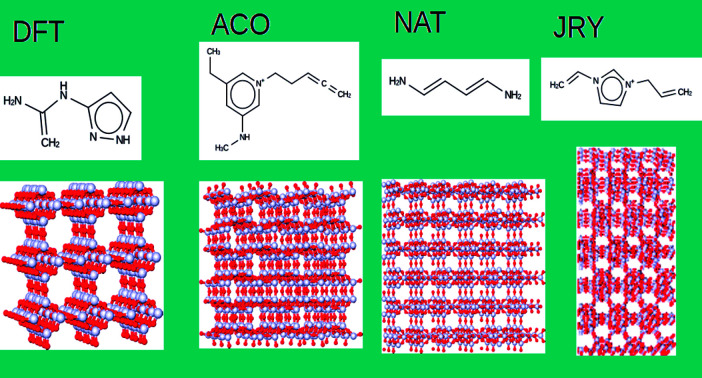

## Introduction

Separation of ethylene–ethane mixtures produced by cracking of naphtha or ethane is an important industrial process.^1^ It is most commonly performed by cryogenic distillation, which is very energy-consuming.^2^ For instance, in the production of ethylene by steam cracking of ethane, 80% of the energy budget is consumed in the heat transfer and separation steps.^3^ Therefore, alternative separation techniques are the subject of intensive research. Zeolites are a class of inorganic nanoporous materials that are widely used in separation by adsorption.^4^ As of today, 248 zeolite frameworks differing in pore size and geometry are known.^5^ This has motivated a number of computational screening studies to identify zeolites that selectively adsorb ethylene *versus* ethane or ethane *versus* ethylene.^6,7^ In the latter study, Shah *et al.* identified a number of promising zeolite frameworks for ethylene–ethane separation using a detailed molecular analysis.^7^ The zeolites identified are predicted to have a high selectivity and adsorption capacity in their all-Si forms. All-Si zeolites are less polar than their Al-containing forms and therefore generally exhibit a lower adsorption enthalpy, which can provide a practical advantage by reducing the regeneration energy needed in the desorption step of the separation.

Synthetic zeolites are typically synthesized by hydrothermal reaction from a suitable Si source, often in the presence of organic structure determining agents (OSDAs).^8,9^ These OSDAs promote the nucleation and growth of zeolites in the reaction mixture, and their structure directing capacity towards a given zeolite framework is correlated with the non-bonding interaction between the OSDA and the zeolite.^10,11^ We have developed a *de novo* design algorithm to computationally design OSDAs for zeolites.^12,13^ We have successfully applied this algorithm to the design and synthesis of novel OSDAs for several zeolite frameworks that led to their subsequent zeolite synthesis.^14–17^ In the present paper, we report our design efforts towards OSDAs for templating all-Si zeolites with the DFT and ACO frameworks for ethylene adsorption and the NAT and JRY frameworks for ethane adsorption. These are the frameworks predicted to be most efficient in the work of Shah *et al.*^7^

## Methods

The *de novo* design program used to discover OSDAs was a genetic algorithm (GA) that generates synthesis routes to molecules that score well in a user-supplied scoring function.^12,13,18^ The molecule generation and the scoring function were separate programs. The genetic algorithm started by randomly generating a population of 100 synthesis routes. A synthesis route consisted of one or several well documented organic reaction steps that operated on a set of commercially available building blocks. To limit the complexity of the synthesis route, the number of synthesis steps was limited to three for generation of the initial population, and to five during the evolution of the initial population. At present, a set of 100 organic chemistry reactions have been implemented into the *de novo* design algorithm. However, a subset of only 61 reactions was used in the present study (*vide infra*). They are summarized in Table SI 5.† The sets of available building blocks were organized as shelves. Four different shelves were used: a set of 69 524 reagents from the Aldrich Market-Select database^19^ with up to ten heavy atoms (denoted as MS10), a set of 597 reagents from the same database with up to five heavy atoms (denoted as MS5), a set of 10 180 reagents from the Sigma-Aldrich building block database^20^ in which the number of rotatable bonds was no larger than one (denoted as ntor_1), and a set of 6983 reagents from the ChemSpace database^21^ with the restriction that each reagent cost no more than 100 USD per gram (denoted as CS100). For each synthesis route, the 2D structure of the resulting reaction product was generated, and this was submitted to the scoring function to obtain its 3D structure and its score, or fitness. This fitness consisted of a vector of scores, as explained below. The values in the score vector were used as binary filters, thresholds, brackets, or values to be minimized. Upon completion of the initial population of the GA, it was divided into Pareto fronts: within each Pareto front, no synthesis route scored better on one score without scoring worse than a synthesis route within the same front. The population was sorted with the more favorable scoring Pareto fronts ranked higher than the less favorable ones. The population was then evolved using one of six genetic operators:

1. Add a reaction step to a synthesis route.

2. Delete a reaction step from a synthesis route.

3. Replace a reagent in a synthesis route by a randomly selected other reagent.

4. Replace a reagent in a synthesis route by a similar selected other reagent.

5. Combine two synthesis routes.

6. Generate a completely new synthesis route.

Operators one through four operated on a single parent synthesis route. Operator five operated on two parent synthesis routes. The parent synthesis routes were selected from the ranked population by tournament selection: two synthesis routes were selected at random, and the higher ranked of the two was retained. The reaction product of the newly generated synthesis route was evaluated by the scoring function, and if its score was better than the worst scoring synthesis route the latter was replaced by it and the population was re-sorted in a Pareto way. Within each Pareto front, the synthesis routes were ranked by the order in which they were generated, with the newer synthesis routes ranked higher than the older ones. The evolution of the GA was continued until a total number of 100 000 synthesis routes had been generated and evaluated. At no point in the GA was it necessary to synchronize the work flow, and therefore it was efficiently run in parallel on a large number of CPUs.

The fitness of a synthesis route consisted of a vector of 2D properties of the reaction product, and of the stabilization energy of the product when fitted into a target zeolite (Table 1). The 2D properties were used as filters to penalize molecules that would be unstable as OSDAs. Thus, the flexibility of the molecules was limited by constraining the number of rotatable bonds. To make the compounds more resilient against the reaction conditions in an eventual zeolite synthesis, no atoms other than C, N, and H, and no triply bonded carbons were allowed. Additionally, the bond distance between two charge centers in a molecule was required to be greater than two, and the ratio of uncharged to charged N atoms was required to be less than or equal to two. It has been observed that most existing effective ODSAs contain a positively charged N atom, with the ratio of C atoms to charged N atoms between 4 and 18.^22^ Therefore, this ratio was also included in the score vector. The 2D properties were either used as binary scores, brackets, or thresholds, as shown in Table 1. If the above listed 2D constraints were met, a low energy conformation of the OSDAs was generated.^23^ A number of OSDA copies were then fitted into the zeolite structure by a combination of a Fourier transform method to determine the optimal translation and random rotation to determine the optimal orientation.^12^ The zeolite structures were obtained from the IZA database^5^ as cif files of the all-silica structures. The zeolite stabilization energy of an OSDA was determined by molecular dynamics simulation of the zeolite–OSDA complex, the original zeolite structure, and the original OSDA.^12^ The stabilization energy was divided by the number of Si atoms in the zeolite.

**Table 1:** Scoring function, score types, and threshold values for the 2D properties in the score vector

Property	Type	Thresholds
Rotatable bonds	Threshold	≤5
Non-C, N, or H atoms	Binary	
Triply bonded C	Binary	
Distance between charge centers	Threshold	≥3
Ratio of total N to charged N	Threshold	≤2
C to charged N	Bracketed	4–14
Stabilization energy in kJ per (mol Si)	Minimize	

The number of OSDAs to be fitted into the zeolite was determined by trial and error for each target zeolite. For some zeolites, the unit cell was expanded along the *c* axis to better accommodate putative OSDAs. It was noted that sometimes after the dynamics simulation, the resulting zeolite structures were severely distorted. Therefore, the Si–O bond lengths and O–Si–O angles were checked, as well as the non-bonded Si–Si or O–O distances. Structures in which the Si–O bonds were 0.5 Å off their value in the optimized IZA structure or in which the O–Si–O bond angles where more than 30° off the tetrahedral angle of 109° were rejected. Also, structures with Si–Si or O–O distances shortened by more than 0.5 Å from their observed minimal distances in the optimized IZA structures were discarded. These thresholds values are summarized in Table 2.

**Table 2:** Angle and distance constraints used to verify zeolite–OSDA complexes

Feature	Threshold
Minimal Si–O distance	1.1 Å
Maximal Si–O distance	2.1 Å
Minimal O–Si–O angle	80°
Maximal O–Si–O angle	140°
Minimal Si–Si distance	2.5 Å
Minimal O–O distance	2.1 Å

To visualize sets of molecules generated by the *de novo* design program in the chemical search space, principal coordinate analysis (PCA)^24^ was applied to distance matrices obtained from the 2-D similarities between molecules. These distance matrices were derived from the Tanimoto similarity coefficients between the molecules calculated from the MACCS fingerprints using openbabel.^25^

## Results

The results of the design efforts are summarized in Tables 3–6. The columns display a reference number to discriminate between different design runs, the number of MD runs, the number of OSDAs with a negative stabilization energy, the number of OSDAs with a stabilization energy within 2 kJ per (mol Si) from the most favorably scoring OSDA in each run, and the identifier, stabilization energy, and 2D structure of the most favorably scoring OSDA.

**Table 3:** Summary of the three most successful design runs on the DFT zeolite, using a single copy of the OSDA. The columns contain an identifier for the run, the number of MD calculations performed during the run, the number of generated OSDAs with a negative stabilization energy, the number of generated OSDAs with a stabilization energy within 2 kJ per (mol Si) from the most favorably scoring OSDA in that run, and the name, stabilization energy, and 2D structure of the most favorably scoring OSDA

Run	Number of MD calculations	Number <0 kJ per (mol Si)	Number within 2 kJ per (mol Si)	Best scoring
DFT 1a	11 229	919	5	Syn030205
				−15.3 kJ per (mol Si)
				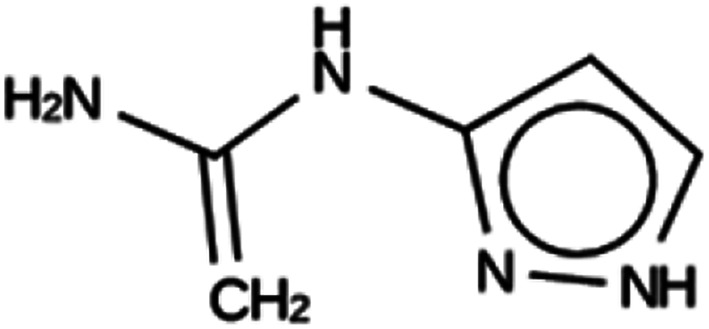
DFT 1b	10 215	847	9	Syn101567
				−14.4 kJ per (mol Si)
				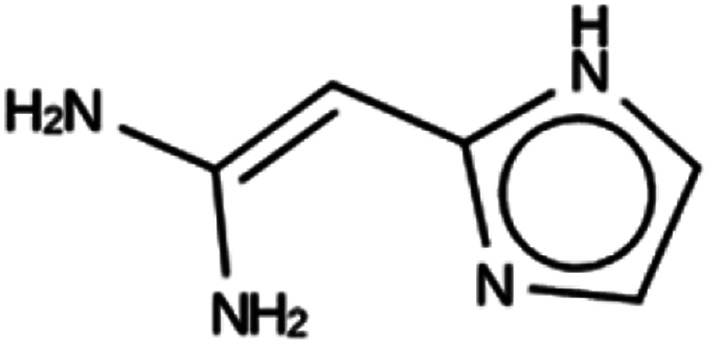
DFT 1c	10 985	951	16	Syn102867
				−13.9 kJ per (mol Si)
				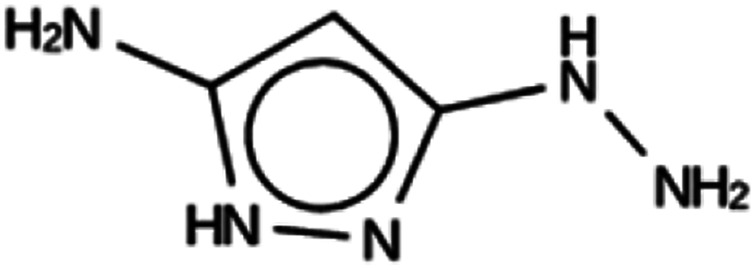

**Table 4:** Summary of the three most successful design runs on the ACO zeolite, using a single copy of the OSDA. The columns contain an identifier for the run, the number of MD calculations performed during the run, the number of generated OSDAs with a negative stabilization energy, the number of generated OSDAs with a stabilization energy within 2 kJ per (mol Si) from the most favorably scoring OSDA in that run, and the name, stabilization energy, and 2D structure of the most favorably scoring OSDA

Run	Number of MD calculations	Number <0 kJ per (mol Si)	Number within 2 kJ per (mol Si)	Best scoring
ACO 1a	6963	128	2	Syn050674
				−7.4 kJ per (mol Si)
				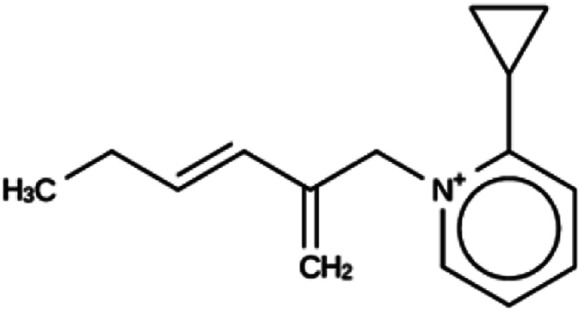
ACO 1b	6858	137	1	Syn080488
				−7.7 kJ per (mol Si)
				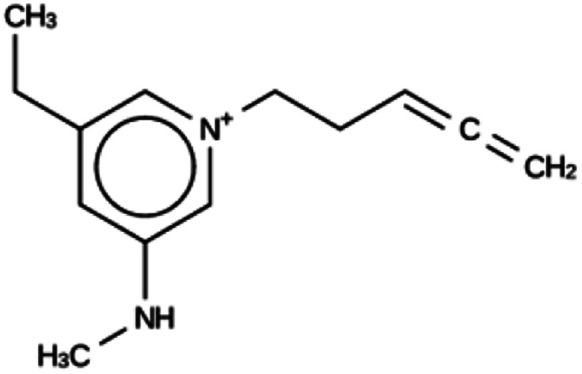
ACO 1c	6755	146	2	Syn030403
				−7.9 kJ per (mol Si)
				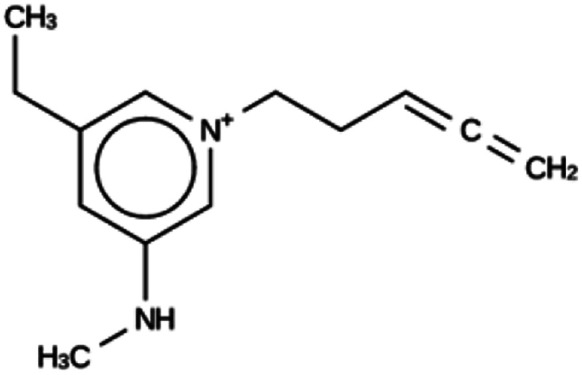

**Table 5:** Summary of the three most successful design runs on the NAT zeolite, using four copies of the OSDA. The columns contain an identifier for the run, the number of MD calculations performed during the run, the number of generated OSDAs with a negative stabilization energy, the number of generated OSDAs with a stabilization energy within 2 kJ per (mol Si) from the most favorably scoring OSDA in that run, and the name, stabilization energy, and 2D structure of the most favorably scoring OSDA

Run	Number of MD calculations	Number <0 kJ per (mol Si)	Number within 2 kJ per (mol Si)	Best scoring
NAT 1a	6185	782	10	Syn044841
				−20.5 kJ per (mol Si)
				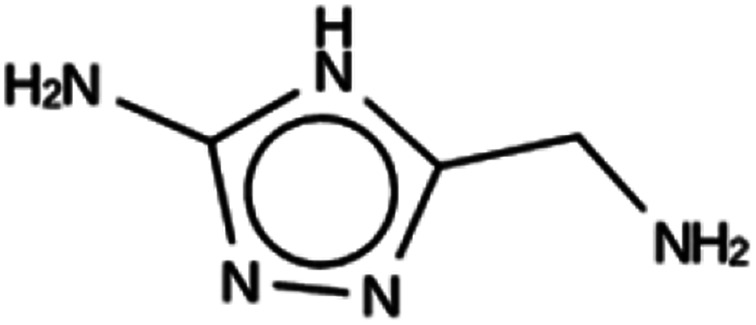
NAT 1b	6152	808	6	Syn123042
				−21.5 kJ per (mol Si)
				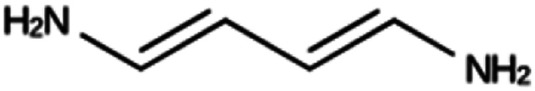
NAT 1c	5990	813	9	Syn020634
				−20.4 kJ per (mol Si)
				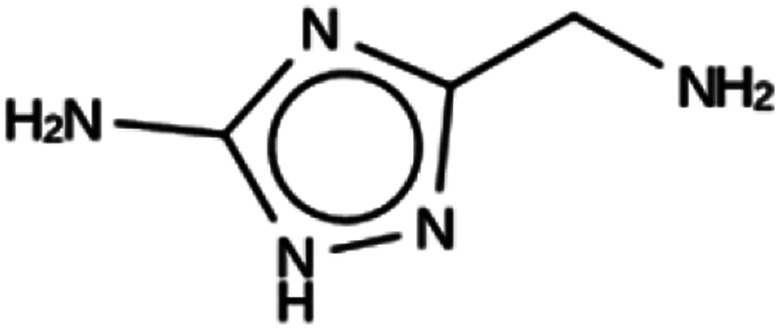

**Table 6:** Summary of the three most successful design runs on the JRY zeolite, using two copies of the OSDA. The columns contain an identifier for the run, the number of MD calculations performed during the run, the number of generated OSDAs with a negative stabilization energy, the number of generated OSDAs with a stabilization energy within 2 kJ per (mol Si) from the most favorably scoring OSDA in that run, and the name, stabilization energy, and 2D structure of the most favorably scoring OSDA

Run	Number of MD calculations	Number <0 kJ per (mol Si)	Number within 2 kJ per (mol Si)	Best scoring
JRY 1a	6964	830	33	Syn051111
				−9.1 kJ per (mol Si)
				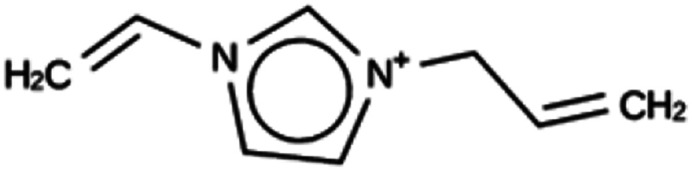
JRY 1b	7001	927	28	Syn036105
				−9.5 kJ per (mol Si)
				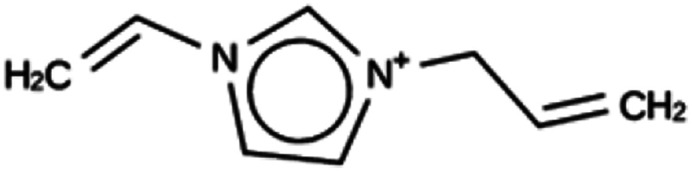
JRY 1c	6522	887	34	Syn007049
				−9.3 kJ per (mol Si)
				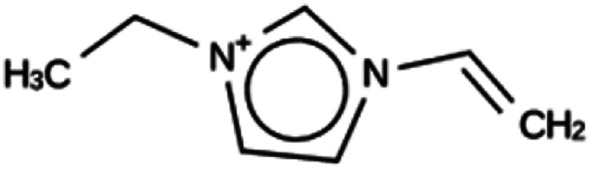

For DFT, no favorably scoring OSDAs were initially discovered. Several design runs were undertaken, with different numbers of OSDA copies, and three different reagent databases (rows 4 through 9 in Table SI 1†). While the majority of known OSDAs are charged, a significant fraction are uncharged. Thus, for DFT we decided to drop the requirement that the OSDAs have a positive charge. This led to favorably scoring OSDAs with stabilization energies below −15 kJ per (mol Si), as shown in Table 3. We also performed two design runs with the smaller CS100 reagent database, but this led to less favorably scoring OSDAs (rows ten and eleven in Table SI 1†).

For ACO, initially two OSDA copies were fitted into the zeolite, resulting in stabilization energies no lower than −4.4 kJ per (mol Si). Upon reducing the number of OSDA copies to one, stabilization energies below −7 kJ per (mol Si) were obtained, as shown in Table 4. The most favorably scoring OSDAs from design runs ACO 1b and ACO 1c are identical.

For NAT, no favorably scoring charged ODSAs were found. Runs with either one or two OSDA copies were unsuccessful, with best scoring OSDAs having stabilization energies no lower than −2.1 kJ per (mol Si) (rows 4 through 7 in Table SI 3†). The NAT structure has an alternative origin in the high symmetry setting. Starting with the *P*1 symmetry version of the structure did not improve the results (rows 8 and 9 in Table SI 3†). As for DFT, however, relaxing the requirement that the OSDAs be charged led to stabilization energies below −5 kJ per (mol Si) with a single OSDA copy fitted into the zeolite (rows 10 through 12 in Table SI 3†). Increasing the number of OSDA copies to two and four further improved this result (columns 13 through 15 and 1 through 3 in Tables SI 3,† and 5). The most favorably scoring OSDAs from design runs NAT_1a and NAT_1c are tautomers of the same molecules.

For JRY, three *de novo* design runs were performed with 2 OSDA copies. This resulted in best scoring molecules with energies below −9 kJ per (mol Si), as shown in Table 6. The most favorably scoring OSDAs from design runs JRY 1a and JRY 1b are identical.

**Fig. 1:**
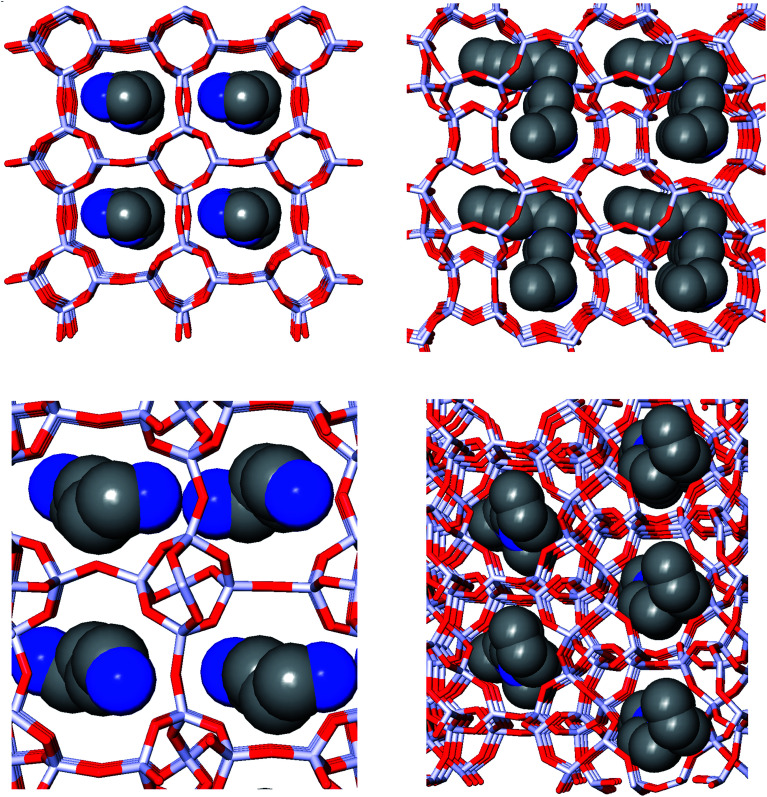
Zeolite–OSDA complexes of the most favorably scoring OSDAs in DFT (upper left), ACO (upper right), NAT (lower left), and JRY (lower right).

Fig. 1 depicts the zeolite–OSDA complexes of the best scoring OSDA for DFT, ACO, NAT, and JRY.

**Fig. 2:**
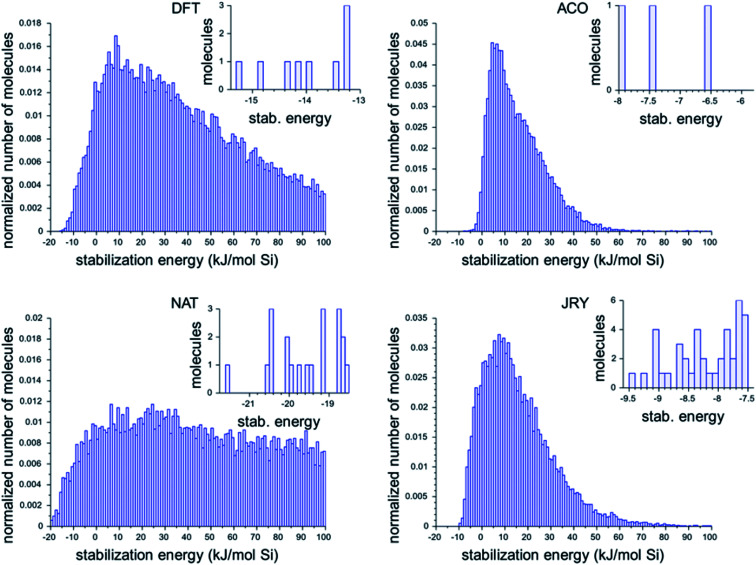
Histograms of the calculated stabilization energies found in the most favorably scoring runs in the four target zeolite frameworks. The large normalized histograms show all unique molecules with a stabilization energy below 100 kJ per (mol Si). The insets show the stabilization energies of the OSDAs with a stabilization energy within 2 kJ per (mol Si) from the best scoring OSDA in all runs for each zeolite.

Fig. 2 shows the histograms of the three best design runs for each framework. The large histograms are the normalized histograms for all unique ODSAs scoring below 100 kJ per (mol Si). The insets present the numbers of OSDAs having a score within 2 kJ per (mol Si) of the best scoring molecule of all runs in each zeolite.

**Fig. 3:**
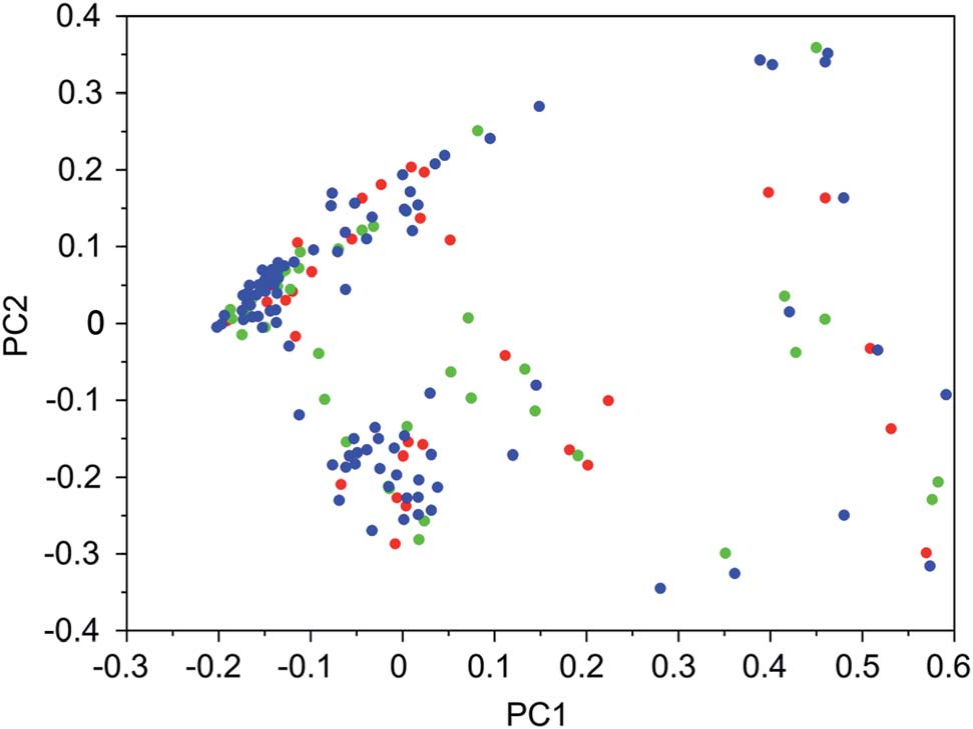
Principal coordinate plot for the 100 best scoring OSDAs designed for DFT. The red, blue, and green data points correspond to runs DFT 1a, DFT 1b, and DFT 1c, respectively. The fractions of variance covered in the two principal coordinates are 0.26 and 0.18.

**Fig. 4:**
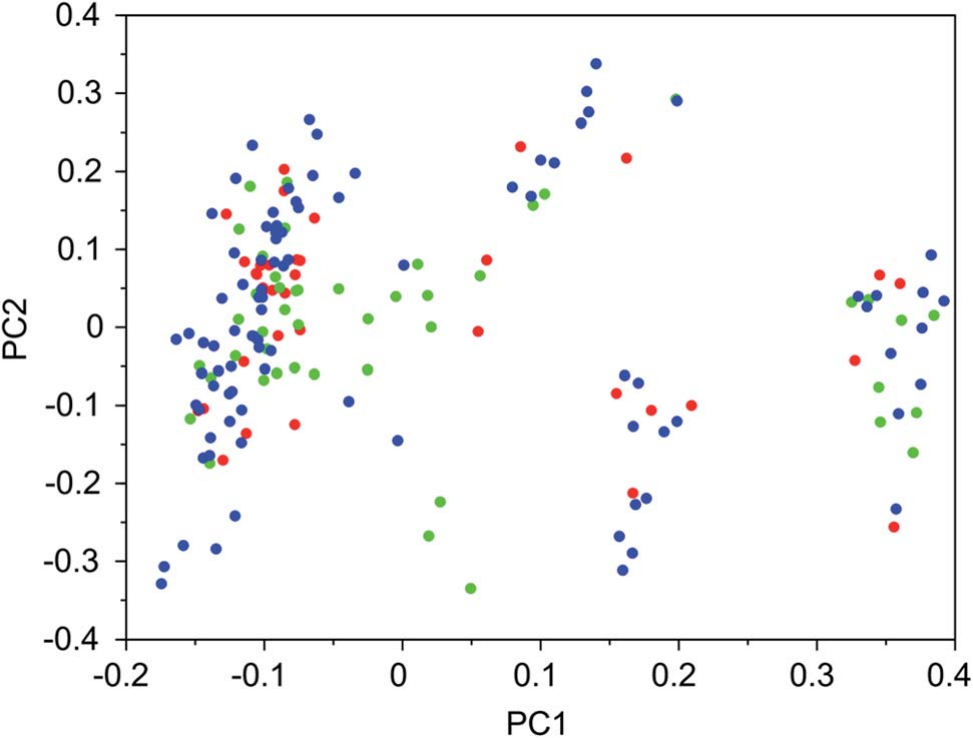
Principal coordinate plot for the 100 best scoring OSDAs designed for ACO. The red, blue, and green data points correspond to runs ACO 1a, ACO 1b, and ACO 1c, respectively. The fractions of variance covered in the two principal coordinates are 0.30 and 0.17.

**Fig. 5:**
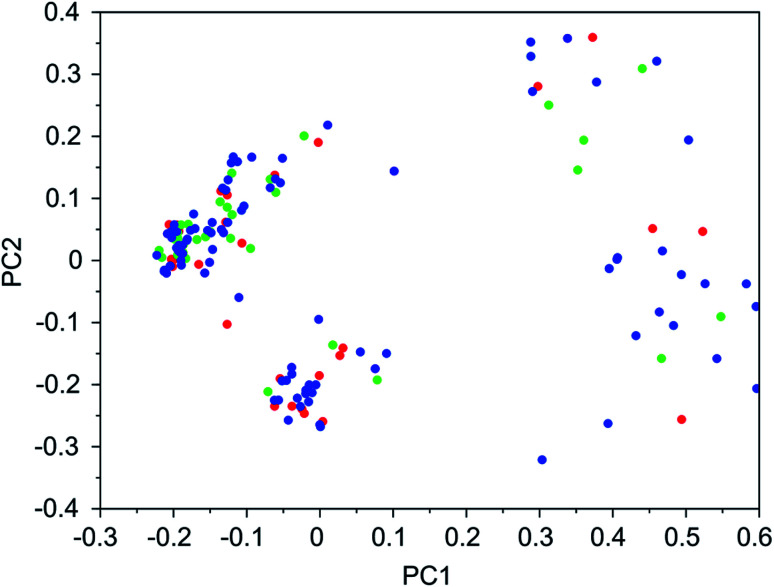
Principal coordinate plot for the 100 best scoring OSDAs designed for NAT. The red, blue, and green data points correspond to runs NAT 1a, NAT 1b, and NAT 1c, respectively. The fractions of variance covered in the two principal coordinates are 0.38 and 0.14.

**Fig. 6:**
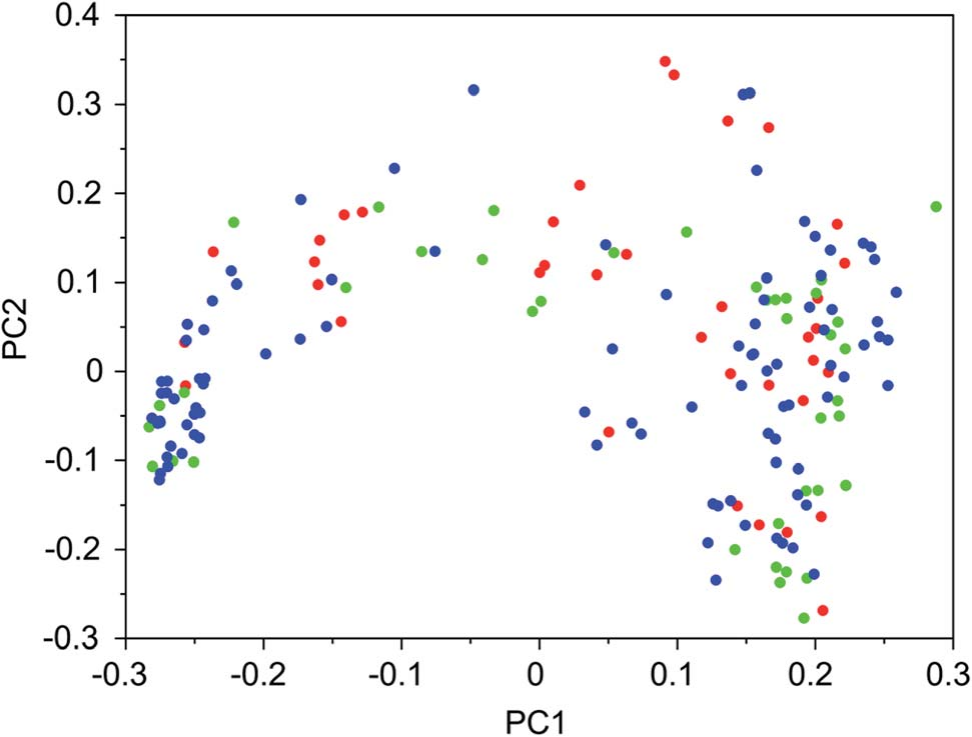
Principal coordinate plot for the 100 best scoring OSDAs designed for JRY. The red, blue, and green data points correspond to runs JRY 1a, JRY 1b, and JRY 1c, respectively. The fractions of variance covered in the two principal coordinates are 0.35 and 0.13.

It was found that in multiple design runs for the same target zeolite, identical OSDAs were often produced. This is illustrated in Tables 7–10, where the sizes of the cross sections between OSDAs scoring below 0 kJ per (mol Si) in the three most productive design runs are listed. To visualize how the GA searches chemical space, principal coordinate plots of the 100 best scoring molecules in the most productive runs are shown in Fig. 3–6.

**Table 7:** Overlap between different OSDAs designed for DFT. Only molecules having a stabilization energy below 0 kJ per (mol Si) were included in the overlap count

	DFT 1a	DFT 1b	DFT 1c
DFT 1a	919	441	473
DFT 1b		847	464
DFT 1c			951

**Table 8:** Overlap between different OSDAs designed for ACO. Only molecules having a stabilization energy below 0 kJ per (mol Si) were included in the overlap count

	ACO 1a	ACO 1b	ACO 1c
ACO 1a	128	54	70
ACO 1b		137	63
ACO 1c			146

**Table 9:** Overlap between different OSDAs designed for NAT. Only molecules having a stabilization energy below 0 kJ per (mol Si) were included in the overlap count

	NAT 1a	NAT 1b	NAT 1c
NAT 1a	782	457	467
NAT 1b		808	474
NAT 1c			813

**Table 10:** Overlap between different OSDAs designed for JRY. Only molecules having a stabilization energy below 0 kJ per (mol Si) were included in the overlap count

	JRY 1a	JRY 1b	JRY 1c
JRY 1a	830	475	465
JRY 1b		927	502
JRY 1c			887

## Discussion

To our knowledge, none of the four zeolites targeted in this study have been reported to be synthesized in an all-silica form, and no OSDAs leading to these all-silica zeolites have been reported. The DFT topology was first discovered in a cobalt phosphate zeolite with ethylenediamine as an OSDA.^26^ A low silica Si/Al material was obtained but was unstable at ambient temperature.^27^ The ACO topology was also discovered in a cobalt phosphate zeolite.^28^ Interestingly, this topology has since been observed in metal organic frameworks designed for CO_2_ storage.^29^ NAT is the framework of the naturally occurring mineral natrolite, with formula Na_2_Al_3_Si_3_O_10_·2H_2_O.^30^ The JRY framework was first identified in a heteroatom-stabilized AlPO_4_ zeolite-analog material.^31^

The choice of all-silica target zeolites DFT and ACO for selected ethylene adsorption, and NAT and JRY for selected ethane adsorption, was based upon the work by Shah *et al.*^7^ In that study, these frameworks scored best in an efficient high-throughput screen using efficient simulation algorithms and accurate molecular force fields. The performance measure of the zeolites for ethane–ethylene separations was the product of the loading of the strongest adsorbing species, *Q*, and the logarithm of the selectivity towards this species, *S*. The latter is defined as *S* = *x*_*i*_/(1 − *x*_*i*_)/[*y*_*i*_/(1 − *y*_*i*_)], where *i* is the more strongly adsorbing species and *x* and *y* are the mole fractions in the zeolite and gas phases, respectively. While the impact of the presence of defects such as silanol or cation impurities is briefly mentioned in ref. 7, the screening was performed on the all-silica materials and hence our OSDA design effort was also directed toward the all-silica zeolites.

The predicted ethylene selectivity of DFT and ACO on one hand, and the ethane selectivity of NAT and JRY on the other hand, has no clear correlation to the structure of the OSDAs designed for these frameworks. This can be rationalized by observing that for effective adsorption, the adsorbents must diffuse into the nanoporous material. An effective OSDA on the other hand, acts as a template during zeolite nucleation and must tightly fit the zeolite nanopores; it is removed by calcination after synthesis.

For the majority of known all-silica zeolites, there is a linear relationship between framework density and calculated lattice energy.^32,33^ This has led to the formulation of a feasibility factor, *θ*, defined as the distance of the data point corresponding to a given zeolite framework from the line of best fit in the energy–density plot. It has been suggested that a value of *θ* ≤ 5 corresponds to feasible zeolites. The values of *θ* for the four zeolites studied here are summarized in Table 11. It can be seen that all four frameworks have a feasibility factor below the suggested threshold.

**Table 11:** Feasibility factors of the four target zeolite frameworks

Framework	Feasibility factor, *θ*
DFT	0.1
ACO	1.1
NAT	3.2
JRY	0.4

For DFT, initially no favorably scoring OSDAs were produced, even when varying parameters such as number of OSDA copies, size of the unit cell, and database of reagents supplied to the *de novo* design algorithm (Table SI 1†). Only when dropping the constraint that a charge center be present in the designed molecules were favorably scoring OSDAs found. All these zeolites separate ethylene and ethane. DFT, ACO, and NAT have main channels that are formed by eight-rings. JRY has main channels that are formed by oblique ten-rings. The size of the maximum sphere than can pass through the structures ranges from 3.65 to 4.4 Å. The size of the maximum sphere than can be included within the structures ranges from 4.52 to 5.1 Å. From these criteria, it is not clear what distinguishes DFT and NAT from ACO and JRY. Only full design runs revealed the requirement that OSDAs designed for the former be uncharged. For the latter targets, charged OSDAs were found in the form of alkylated five- or six-ring heterocycles. Apparently, the inclusion of a methyl or larger group even into a five-ring hetero cycle produced molecules that are too large to be fitted into the channels and channel sections of DFT and NAT. Although the introduction of charge centers in OSDAs increases solubility in a zeolite synthesis medium, many examples exist of successful uncharged OSDAs. Thus, the designed molecules may be effective OSDAs.

Two design runs with a different, and smaller, reagent database generated less favorably scoring OSDAs (rows ten and eleven in Table SI 1†). Thus, limiting the number of available reagents removed the potential for finding these higher scoring OSDAs in the chemical search space. Increasing the number of available reagents allows the *de novo* design algorithm to find more optimal solutions, using its ability to effectively explore larger chemical search spaces.

OSDAs targeted towards ACO were found by fitting a single OSDA copy into the framework. As the lowest stabilization energies were less favorable than for the DFT, NAT, and JFY OSDAs, a larger number of design runs was carried out (Table SI 2†). Eventually, a molecule with a stabilization energy of −7.9 kJ per (mol Si) was found, as shown in the ACO 1c entry in Table 3. The same molecule was the most favorably scoring OSDA in run ACO 1b, where the calculated stabilization energy was −7.7 kJ per (mol Si). The discrepancy be these two numbers is due to the stochastic nature of the MD procedure.

For NAT, as for DFT, the introduction of even small alkyl groups to the generated molecules so as to produce a positive charge center on the nitrogen atom precluded the design of favorably scoring OSDAs. Relaxing the charge requirement produced favorably scoring OSDAs. The NAT unit cell is characterized by four channels, formed by rings of eight T centers, along the 001 direction (Fig. 1, bottom left). Introducing one, two, and four OSDA copies generated stabilization energies of approximately −5, −10 and −20 kJ per (mol Si) (Tables SI 3† and 5); thus, the total stabilization energy grew linearly with the number of OSDA copies until all four channels of NAT were occupied.

For JRY, two OSDA copies were fitted into the zeolite framework to obtain stabilization energies around −9.5 kJ per (mol Si), as shown in the JRY 2b entry in Table 6. It may be noted that the most favorably scoring OSDAs in design runs JRY 1a and JRY 1b were identical, and very similar to the most favorably scoring OSDA in design run JRY 1c.

Fig. 1 depicts the structures of the zeolite–OSDA complexes of the best scoring OSDAs in each of the four target zeolites. The largest channels of DFT, ACO, and NAT are composed of eight-ring T-centers, while the main channel of JRY is composed of ring of ten T-centers. It can be seen that the designed OSDAs occupy all channels in the unit cell. In order to achieve this, for DFT and ACO, a single OSDA copy was required, while for NAT and JRY two and four OSDA copies were required, respectively.

The histograms in Fig. 2 show that there is considerable difference in the most favorable stabilization energies that have been obtained for the different zeolite targets, and also in the numbers of favorably scoring molecules that have been generated. The most favorably scoring OSDAs are found for the NAT framework. The largest number of favorably scoring OSDAs were found for JRY, with a total number of 56 molecules scoring within 2 kJ per (mol Si) from the lowest stabilization energy of −9.5 kJ per (mol Si) found.

Tables 7–10 show that there is considerable overlap between repeated runs directed towards the same frameworks. Roughly half of the molecules in any pair of runs for a given zeolite overlapped. This is visualized in Fig. 3–6, were it can be seen that different runs explore the chemical space in a largely similar way.

## Conclusion

We have designed OSDAs for four of the zeolites predicted to be most promising for energy-efficient separation of ethylene from ethane. For each zeolite, we designed multiple OSDAs to direct their synthesis. For the ethylene-selective DFT, the OSDAs have stabilization energies within 3 kJ per (mol Si) of the lowest reported values to date for any zeolite. For the ethane-selective NAT, the OSDAs have stabilization energies lower than any reported values to date for any zeolite. These OSDAs may enable the synthesis of zeolites that lead to the construction of more energy-efficient ethylene–ethane separation processes.

## Conflicts of interest

Michael W. Deem is a consultant for the petrochemical industry in the area of zeolites. This relationship did not affect the design or outcome of the present research.

## Supplementary Material

RA-010-D0RA02896G-s001

RA-010-D0RA02896G-s002

RA-010-D0RA02896G-s003

RA-010-D0RA02896G-s004

RA-010-D0RA02896G-s005
