# Supplementary material for: Design of organic structure directing agents to guide the synthesis of zeolites for the separation of ethylene–ethane mixtures
Source: RSC Adv. 2020 May 27;10(34):20313–21. doi: 10.1039/d0ra02896g (PMC9054118; doi:10.1039/d0ra02896g)
Supplement: RA-010-D0RA02896G-s005 [file RA-010-D0RA02896G-s005.pdf]

Electronic Supplementary Material (ESI) for RSC Advances.
This journal is © The Royal Society of Chemistry 2020

Supporting Information

Table SI 1: Design runs for DFT. The columns contain an identifier for the run, the number of OSDA copies fitted into the zeolite, the reference to the reagent database used for the run, the number of MD calculations performed during the run, the number of generated OSDAs with a negative stabilization energy, the number of generated OSDAs with a stabilization energy within 2 kJ/(mol Si) from the most favorably scoring OSDA in each run, and the name, stabilization energy, and 2D structure of the most favorably scoring OSDA. For the DFT 1 and DFT 7 design runs, the requirement that a positive charge center be present in the OSDA was dropped. All molecules in the other design runs did have a positive charge center.

DFT run	OSDAs	shelf	MD	Number <0	Number within 2 kJ/(mol Si)	Best
DFT 1a uncharged	1	MS10	11229	919	5	Syn030205<br>-15.3 kJ/(mol Si)<br>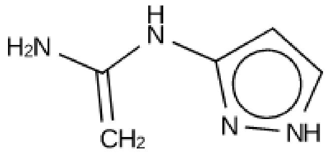
DFT 1b uncharged	1	MS10	10215	847	9	Syn101567<br>-14.4 kJ/(mol Si)<br>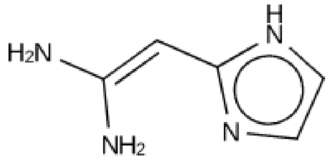
DFT 1c uncharged	1	MS10	10985	951	16	Syn102867<br>-13.9 kJ/(mol Si)<br>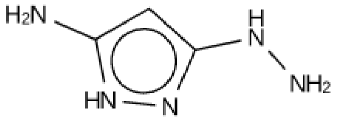

DFT run	OSDAs	shelf	MD	Number <0	Number within 2 kJ/(mol Si)	Best
DFT 2a charged	1	MS10	3954	3	3	Syn145277<br>-1.7 kJ/(mol Si)<br>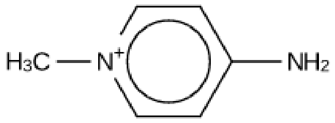
DFT 2b charged	1	MS10	8616	11	2	Syn031296<br>-3.5 kJ/(mol Si)<br>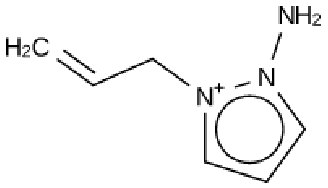
DFT 3 charged	1	ntor_1	2732	5	4	Syn008292<br>-2.1 kJ/(mol Si)<br>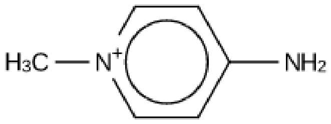
DFT 4 charged	1	MS05	1252	37	37	Syn032690<br>-2.1 kJ/(mol Si)<br>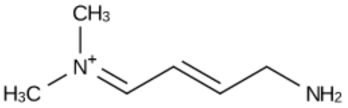

DFT run	OSDAs	shelf	MD	Number <0	Number within 2 kJ/(mol Si)	Best
DFT 5 charged	3	MS10	6002	7	3	Syn066720<br>-2.7 kJ/(mol Si)<br>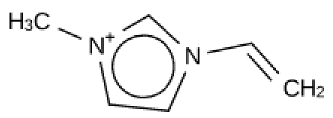
DFT 6 charged	4	MS10	4275	2	2	Syn027565<br>-1.5 kJ/(mol Si)<br>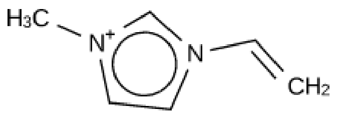
DFT 7a uncharged	1	CS100	3974	107	4	Syn007797<br>-9.5 kJ/(molSi)<br>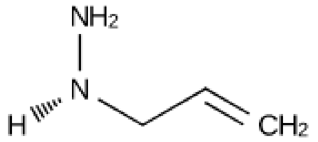
DFT 7b uncharged	1	CS100	3541	97	4	Syn052193<br>-9.4 kJ/(molSi)<br>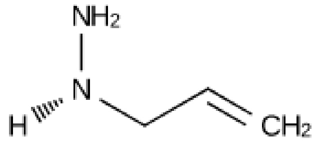

Table SI 2: Design runs for ACO. The columns contain an identifier for the run, the number of OSDA copies fitted into the zeolite, the reference to the reagent database used for the run, the number of MD calculations performed during the run, the number of generated OSDAs with a negative stabilization energy, the number of generated OSDAs with a stabilization energy within 2

kJ/(mol Si) from the most favorably scoring OSDA in each run, and the name, stabilization energy, and 2D structure of the most favorably scoring OSDA.

ACO run	OSDAs	shelf	MD	Number <0	Number within 2 kJ/(mol Si)	Best
ACO 1a	1	MS10	6963	128	2	Syn050674<br>-7.4 kJ/(mol Si)<br>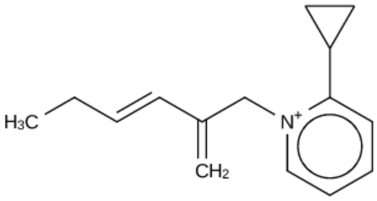
ACO 1b	1	MS10	6858	137	1	Syn080488<br>-7.7 kJ/(mol Si)<br>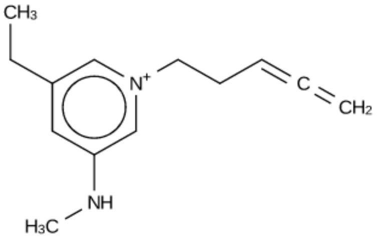
ACO 1c	1	MS10	6755	146	2	Syn030403<br>-7.9 kJ/(mol Si)<br>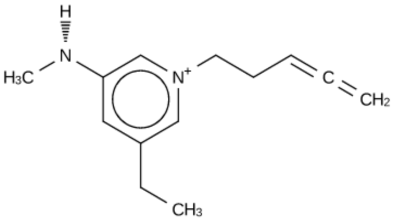

ACO run	OSDAs	shelf	MD	Number <0	Number within 2 kJ/(mol Si)	Best
ACO 1d	1	MS10	6546	132	4	Syn083813<br>-7.2 kJ/(mol Si)<br>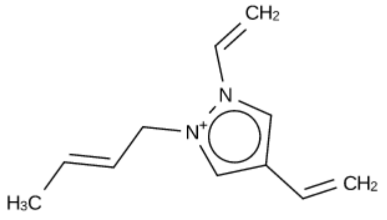
ACO 1e	1	MS10	7192	123	9	Syn061370<br>-6.0 kJ/(mol Si)<br>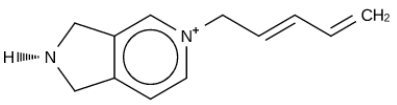
ACO 1f	1	MS10	6667	129	1	Syn024815<br>-7.1 kJ/(mol Si)<br>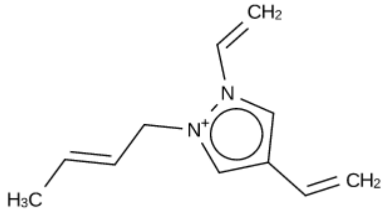

Table SI 3: Design runs for NAT. The columns contain an identifier for the run, the number of OSDA copies fitted into the zeolite, the reference to the reagent database used for the run, the number of MD calculations performed during the run, the number of generated OSDAs with a negative stabilization energy, the number of generated OSDAs with a stabilization energy within 2 kJ/(mol Si) from the most favorably scoring OSDA, and the name, stabilization energy, and 2D structure of the most favorably scoring OSDA in each run. The first column also indicates which zeolite symmetry is used, and whether the OSDA are charged or uncharged.

NAT run	OSDAs	shelf	MD	Number <0	Number within 2 kJ/(mol Si)	Best

NAT run	OSDAs	shelf	MD	Number <0	Number within 2 kJ/(mol Si)	Best
NAT 1a P1 uncharged	4	MS10	6185	782	10	Syn044841 -20.5 kJ/(mol Si) 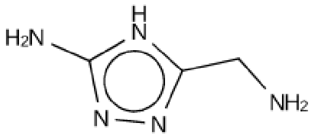
NAT 1b P1 uncharged	4	MS10	6152	808	6	Syn123042 -21.5 kJ/(mol Si) 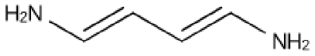
NAT 1c P1 uncharged	4	MS10	5990	813	9	Syn020634 -20.4 kJ/(mol Si) 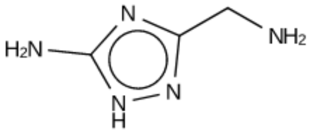
NAT_2a charged	1	MS10	7070	25	25	Syn125615 -1.3 kJ/(mol Si) 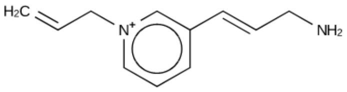
NAT_2b charged	1	MS10	6190	37	21	Syn010154 -1.3 kJ/(mol Si) 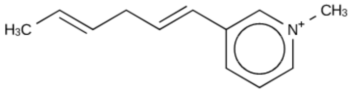

NAT<br>run	OSDAs	shelf	MD	Number<br><0	Number within<br>2 kJ/(mol Si)	Best
NAT_2c<br>charged	1	MS10	6684	17	17	Syn070894<br>-1.3 kJ/(mol Si)<br>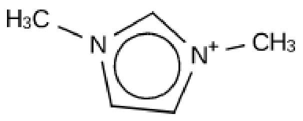
NAT_3a<br>charged	2	MS10	6592	4	4	Syn157731<br>-2.1 kJ/(mol Si)<br>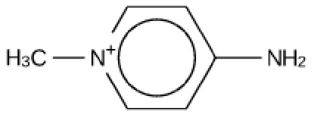
NAT_4a<br>P1<br>charged	1	MS10	7079	22	22	Syn061573<br>-1.7 kJ/(mol Si)<br>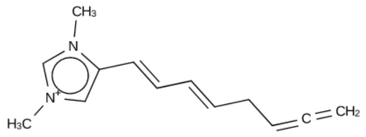
NAT_4b<br>P1<br>charged	1	MS10	7149	25	25	Syn018329<br>-1.2 kJ/(mol Si)<br>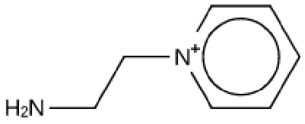
NAT_5a<br>P1<br>uncharged	1	MS10	15106	1774	128	Syn004462<br>-5.5 kJ/(mol Si)<br>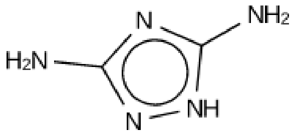

NAT run	OSDAs	shelf	MD	Number <0	Number within 2 kJ/(mol Si)	Best
NAT_5b P1 uncharged	1	MS10	14755	1738	124	Syn050548<br>-5.6 kJ/(mol Si)<br>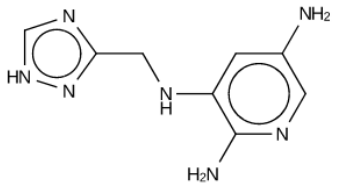
NAT_5c P1 uncharged	1	MS10	14058	1656	194	Syn047941<br>-5.2 kJ/(mol Si)<br>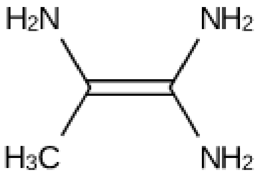
NAT_6a P1 uncharged	2	MS10	10506	10506	1758	Syn010950<br>-10.9 kJ/(mol Si)<br>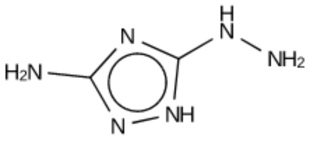
NAT_6b P1 uncharged	2	MS10	10225	10225	1777	Syn078845<br>-10.0 kJ/(mol Si)<br>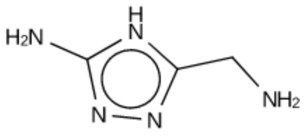

NAT run	OSDAs	shelf	MD	Number <0	Number within 2 kJ/(mol Si)	Best
NAT_6c P1 uncharged	2	MS10	10643	10643	1771	Syn165261<br>-10.1 kJ/(mol Si)<br>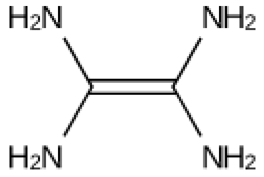

Table SI 4: Design runs for JRY. The columns contain an identifier for the run, the number of OSDA copies fitted into the zeolite, the reference to the reagent database used for the run, the number of MD calculations performed during the run, the number of generated OSDAs with a negative stabilization energy, the number of generated OSDAs with a stabilization energy within 2 kJ/(mol Si) from the most favorably scoring OSDA in each run, and the name, stabilization energy, and 2D structure of the most favorably scoring OSDA.

JRY run	OSDAs	shelf	MD	Number <0	Number within 2 kJ/(mol Si)	Best
JRY 1a	2	MS10	6964	830	33	Syn051111<br>-9.1 kJ/(mol Si)<br>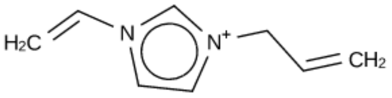
JRY 1b	2	MS10	7001	927	28	Syn036105<br>-9.5 kJ/(mol Si)<br>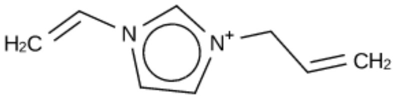

JRY run	OSDAs	shelf	MD	Number <0	Number within 2 kJ/(mol Si)	Best
JRY 1c	2	MS10	6522	887	34	Syn007049<br>-9.3 kJ/(mol Si)<br>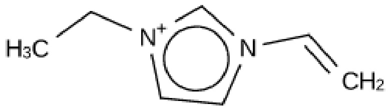

Table SI 5: The organic synthesis reactions used by the de novo *design program to generate OSDAs.*

**Mnemonic**	**Reactants**	**Product**	**Description**
AMIDEFROMACID	$R_1C{=}OOH$, R_2NH_2	R_1-NHC=O-R_2	Amide from acid and aliphatic amine
REDUCECYAN	RC#N	R-CNH_2	Red.Am. Fully reduce CN to amine
WITTIG	R_1CXR_2, $R_3C{=}OR_4$	$R_{1,2}C{=}CR_{3,4}$	Wittig alkene synthesis
NH2FROMNITRO	RNO_2	RNH_2	Reduce nitro group
AMINEFROMAMIDE	$R_1C{=}ONR_{2,3}$	$R_1CH_2NR_{2,3}$	Red.Am. Reduce amide group
AMINEFROMACID	RC=OOH	RNH_2	Schmidt Reaction, reduce acid group
AMINEFROMALDOKETO	$R_1C{=}OR_2$	$R_1CHNH_2R_2$	Reductive amination from carbonyl
FCACYLATION	Aryl, $R_2C{=}OX$	Ar1C=OR_2	Friedel-Crafts acylation
DEOXYALDOKETO	$R_1C{=}OR_2$	$R_1CH_2R_2$	Aldol/Ketone deoxgenation
REDALDOKETO	$R_1C{=}OR_2$	R_1CHOHR_2	Reduction of aldol/ketone to alchohol
GABRIEL	RX	RNH_2	Alkyl-NH2 from halide
ALKYNECOUPLING	R_1C#CH, R_2C#CH	R_1C#CC#CR_2	Alkyne coupling
APPEL	ROH	RX	Appel reaction
BARTOLIINDOLE	R_1NO_2Phe, $XR_2C{=}CR_3$	$R_{1,2,3}$ Indole	Bartoli indole synthesis
BUCHWALDHARTWIG	ArX, R_1NHR_2	$ArNR_{1,2}$	Buchwald Hartwig coupling
ALKENETOALKANE	$R_{1,2}C{=}CR_{3,4}$	$R_{12}CHCHR_{34}$	Reduction of alkene
COREYFUCHS	RHC=O	RC□CH	Corey-Fuchs alkyne synthesis
ALKYNETOALKENE	R_1C□CR_2	$R_1HC{=}CHR_2$	Reduction of alkyne to alkene
ALKYNETOALKANE	R_1C□CR_2	$R_1H_2CCH_2R_2$	Reduction of alkyne to alkane
ALKYNESN2	R_1C□CH, R_2X	R_1C□CR_2	SN2 displacement of alkyne
OHTOBR	ROH	RBr	Bromide synthesis
OHTOI	ROH	RI	Iodide synthesis
BENZYLTOCHLORIDE	$ArCH_3$	$ArCH_2Cl$	Benzyl halogenization
BENZYLTOBROMIDE	$ArCH_3$	$ArCH_2Br$	Benzyl halogenization
HALOTOCYANIDE	RX	RC□N	Cyanide from halide
SONOGASHIRA	R_1C#CH, R_2X	R_1C#CR_2	Sonogashira coupling
LAROCKINDOLE	$ArINHR_1$,R_2C□CR_3	R_{123}indol	Larock indole synthesis
SUZUKI	borate, halogenide	Alkyl/aryl$_1$-Alkyl/aryl$_2$	Suzuki coupling
NHALKYLATION	aromatic N-H, RX	ArN-R, ArN = imidazole, ...	Alkylation of arom. N with Grignard
FISCHERINDOLE	Hydrazine, carbonyl	indole	Fischer indole synthesis
MENSHUTKIN	R1R2R3N, R4X	Quaternary amine	Menshutkin reaction
SKRAUP	anyline with alpha-CH	quinoline	Skraup reaction
SUZUKIMIYAURA	2x Alkyl/aryl -X	Alkyl/aryl$_1$-Alkyl/aryl$_2$	Suzuki-Miyaura reaction

**Mnemonic**	**Reactants**	**Product**	**Description**
ESCHWEILERCLARKE	prim./sec. amine	methyl-substituted amine	Eschweiler-Clarke reaction
PYRIMIDINIUM	pyridine, SN2-halogenide	pyrimidinium cation	alkylated pyridine (pyridinium ion)
ALKYLATEN	amine, MeI or EtI	methyl or ethyl amine	fully methylate or ethylate an amine
KNORRPYRAZOLE	RC(=O)CC(=O)R, Hydrazin	Pyrazole	Knorr Pyrazole synthesis
ALKYLATENP	aromatic N, SN2 halegonide	N-alklated aromatic heterocycle	Alkylation of aromatic nitrogen
CXBORYLATION	prim./sec. halogenide	alkyl-$B(OH)_2$	Boronation of halogenide
ALKYLATESECAM	sec. amine	prim./sec. halogenide	Alkylation of sec. amine
ALKYLATENPORNPH	aromatic N or NH	prim./sec. halogenide	Alkylation of aromatic amine
PYRIDINETOC	pyridine	phenyl equivalent	Replace pyridine with phenyl
